# Chronic Diseases of the Joints

**Published:** 1907-11-16

**Authors:** 


### Chronic Diseases of the Joints.

The discussion of the nature and treatment of chronic diseases of the joints is a stock subject for medical societies and congresses. Again and again it figures on the programme, and for the most part the same speakers tell us very much the same things that they have announced on previous occasions. It may be hoped that their industry and eloquence secure an appropriate reward. A considerable part of the discussion usually turns on the question of the terms to be employed, and "rheumatic gout," "rheumatoid arthritis," "osteo-arthritis," and the rest, all have their sponsors and advocates. Yet it may be doubted whether this part of the controversy contributes anything either to scientific medicine or to the problems of actual practice; whether, indeed, it does not succeed rather in darkening counsel, for certainly it includes many "words without knowledge." Further, it is fair to contend that this portion of the discussion is not only useless, but entirely unnecessary. All must admit that the ætiology of the clinical conditions in question is extremely obscure and uncertain. Some authorities may "think" this, and others may "hold" that, but none of them really knows or is prepared to advance more than a personal impression in his defence. In these circumstances it would seem unjustifiable to employ, as descriptive terms, names which imply a knowledge of causation. Still again, the use of such terms may be condemned as a matter of general principle. They are bad because they involve recognition of particular theories, and even well-established theories sometimes disappear under the tide of advancing knowledge, while names based on them remain to confuse and agitate succeeding generations. This has been a realised experience in more than one department of medical activity, and it bids fair to be repeated in connection with chronic disease of the joints. "Chronic arthritis" or "chronic multiple arthritis" describes the facts, avoids controversy about names, and leaves the field open for the legitimate operation of new discoveries. With such terms, therefore, our present disputants would do well to be content.

